# Author Response to Comment on: Laboratory Measurement and Analysis of the Deteriorated Layer Permeability Coefficient of Soil-Cement Deteriorated in a Saline Environment

**DOI:** 10.3390/ma13010207

**Published:** 2020-01-03

**Authors:** Qing Jin, Xinzhuang Cui, Junwei Su, Tu Lu, Jieru Wang, Ruonan Han

**Affiliations:** School of Civil Engineering, Shandong University, Jinan 250061, China; jinqing@sdu.edu.cn (Q.J.); sxsujunwei@163.com (J.S.); lutu1995@foxmail.com (T.L.); wjr190420@163.com (J.W.); 201834793@sdu.edu.cn (R.H.)

**Keywords:** deteriorated layer, permeability coefficient, formulates, correction

## Abstract

The authors thank Rui Neves for his discussions related to our work. Errors in the formula have been corrected as suggested by the discusser and data in the article have also been revised.

## 1. Introduction

The authors thank Rui Neves for his discussions related to our work on the deteriorated layer permeability coefficient of soil–cement deteriorated in a saline environment. Based on the discussions with Prof. Neves, some formulates in the paper have been corrected [1]. We have responded in the following aspects.

## 2. Reply

The first response is regarding the lapse in the development of the formula to compute the permeability coefficient in a specimen with different media. We found that Equations (7)–(10) in original paper were wrong due to some errors in the calculation procedure. In the original paper, Equation (7) in original paper is achieved after substituting Equation (6) in original paper in Equation (4) in original paper, which are shown below.

(1) $$k_{d}=\frac{2k_{c}k_{m}d}{k_{m}H-k_{c}H_{m}}$$

(2) $$k_{m}=k_{0}\left(1-R_{a}\right)+k_{d}R_{a}$$

where *k_d_* is the permeability coefficient of deteriorated layer; *k_c_* is the equivalent permeability coefficient of the entire deteriorated specimen; *k_m_* is the equivalent permeability coefficient of the middle section of the specimen; *H* is the total height of the specimen; *H_m_* is the height of the middle section; *k_0_* is the permeability coefficient of the internal non-deteriorated region of the soil-cement; *d* is the deterioration depth of the cement–soil; and *R_a_* is the cross-sectional area deterioration rate of the soil–cement specimen.

The relationship between *H*, *H_m_*, and *d* is

*H* = *H_m_* + 2 *d* (3)

The mistakes occurred when we, in our paper, after substituting Equation (6) in Equation (4), divided the resulting expression by *H*, to introduce the *R*_h_ term. In our paper, we defined *R*_h_ = *d*/*H*. However, the ratio *H_m_*/*H* was amiss and also taken as *R*_h_ in the calculation, causing errors in our original results. We are grateful to Prof. Neves for pointing out these problems. As the second response is also about this formula, the correct formula is shown below.

Second, the discusser points out that our paper adopted an approach where the mass flow in a homogeneous layer is homogeneous, regardless of the eventual heterogeneities in other layers previously crossed by mass. We have checked our paper and believe that the inaccurate equation might refer to Equation (5) in original paper, which is

(4) $$Q_{m}=k_{m}i_{m}A=k_{0}i_{m}\left(A-A_{d}\right)+k_{d}i_{m}A_{d}$$

According to the suggestions made by Prof. Neves, the *k*_0_ in Equation (4) is supposed to be replaced by *k*_m,SA_, which is the equivalent permeability coefficient of the materials in SA. *k*_m,SA_ is calculated as

(5) $$k_{m,SA}=\frac{d+H_{m}+d}{\frac{d}{k_{d}}+\frac{H_{m}}{k_{0}}+\frac{d}{k_{d}}}$$

Equation (5) in origin paper should be

(6) $$Q_{m}=k_{m}i_{m}A=k_{m,SA}i_{m}\left(A-A_{d}\right)+k_{d}i_{m}A_{d}$$

Equation (4) in origin paper is calculated as

(7) $$k_{d}=\frac{2k_{c}k_{m}d}{k_{m}H-k_{c}H_{m}}$$

By substituting Equations (5) and (6) into Equation (7), an equation equivalent to Equation (10) in the comment can be achieved. Equations (8)–(10) in original text is supposed to be replaced as

(8) $$\;\;K_{d}=\frac{A+\surd B}{2R_{a}\left(1-2R_{h}\right)}$$

(9) $$A=K_{c}+K_{0}R_{a}-K_{0}-2K_{c}R_{h}-2K_{0}R_{a}R_{h}$$

(10) $$B=K_{0}^{2}{\left(1-R_{a}+2R_{a}R_{h}\right)}^{2}+2K_{0}K_{C}\left(R_{a}-1+2R_{a}R_{h}\right)\left(1-2R_{h}\right)\;+K_{c}^{2}{\left(1-2R_{h}\right)}^{2}$$

**Figure 1:**
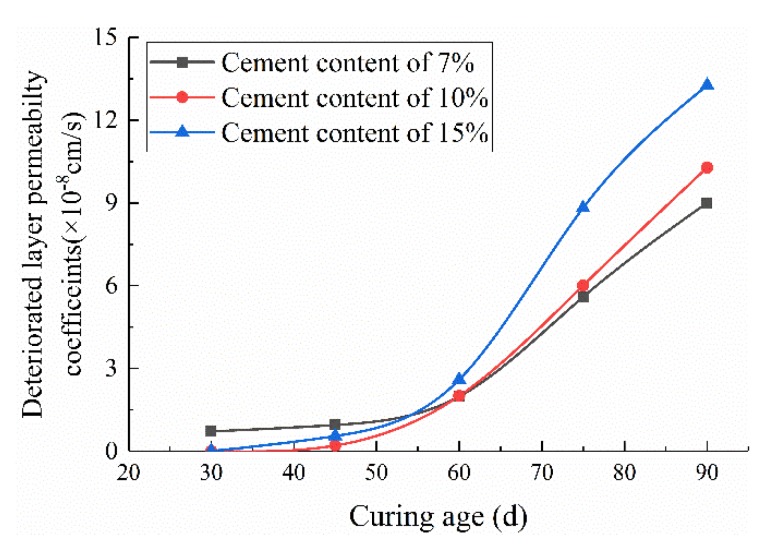
Development of the deteriorated layer permeability coefficients.

In Figure 1, the values of *k_d_* calculated by Equations (8)–(10) are shown.

Finally, as Equations (8)–(10) in origin paper have been revised, the function to model the evolution of the permeability coefficient of a deteriorated part of soil–cement also needs to be revised. According to Equations (8)–(10) the parameters presented in Table 1 can be obtained.

## Figures and Tables

**Table 1:** Fitting parameters.

Cement Content	*k_i_* (×10^−8^ cm/s)	*k_u_* (×10^−8^ cm/s)	*t_c_* (d)	*p*
7%	0.69	12.66	78.90	7.31
10%	0.04	13.81	75.92	7.42
15%	0.03	14.16	75.54	8.97
